# An inter-platform repeatability study investigating real-time amplification of plasmid DNA

**DOI:** 10.1186/1472-6750-5-15

**Published:** 2005-05-25

**Authors:** Carol E Donald, Fizza Qureshi, Malcolm J Burns, Marcia J Holden, Joseph R Blasic, Alison J Woolford

**Affiliations:** 1 Bio-Molecular Innovation Team, LGC Ltd, Queens Road, Teddington, Middlesex TW11 0LY, UK; 2 Bioprocess Measurement Group, National Institute of Standards and Technology (NIST), 100 Bureau Drive, Mail Stop 8312, Gaithersburg, MD 20899-8312, USA

## Abstract

**Background:**

The wide variety of real-time amplification platforms currently available has determined that standardisation of DNA measurements is a fundamental aspect involved in the comparability of results.

Statistical analysis of the data arising from three different real-time platforms was conducted in order to assess inter-platform repeatability. On three consecutive days two PCR reaction mixes were used on each of the three amplification platforms – the LightCycler^®^, ABI PRISM^® ^7700 and Rotor Gene 3000™. Real-time PCR amplification using a fluorogenic 5' exonuclease assay was performed in triplicate on negative controls and DNA plasmid dilutions of 10^8^–10^2 ^copies to give a total of 24 reactions per PCR experiment.

**Results:**

The results of the statistical analyses indicated that the platform with the most precise repeatability was the ABI PRISM^® ^7700 when coupled with the FastStart PCR reaction mix. It was also found that there was no obvious relationship between plasmid copy number and repeatability. An ANOVA approach identified the factors that significantly affected the results, in descending order of magnitude, as: plasmid copy number, platform, PCR reaction mix and day (on which the experiment was performed).

**Conclusion:**

In order to deliver useful, informative genetic tests, standardisation of real-time PCR detection platforms to provide repeatable, reliable results is warranted. In addition, a better understanding of inter-assay and intra-assay repeatability is required.

## Background

A diverse range of applications that impact on clinical diagnosis and prognosis rely on the real-time detection and quantification of Polymerase Chain Reaction products such as bacterial diagnostics [1,2], viral load [3-6], cancerous cell burden [7,8] and parasite quantification [9]. Each particular assay dictates the requirement for instrument capacity, data reproducibility and run length times. There is an expansive collection of different fluorescent chemistries and instruments available for real-time amplification reactions [10-12]. These platforms represent a technological advance from the traditional thermal cycler and typically offer faster, smaller (reduced volume) and more (high-throughput) reactions. Whilst the number of instruments with different software and fluorescent dyes is considerable, the obvious drawback is that the resultant data may not be repeatable or even directly comparable between instruments or even within instruments on different runs. A statistically appropriate number of samples and runs need to be conducted in order to have confidence in the results or to assign mathematical significance to any of the factors affecting real-time amplification. In addition, there is a shortage of reliable, universal DNA standards at present. Increasing diversity through technological advances in DNA quantitation instruments and software may further challenge the standardisation of results. It should be ensured that proper quality controls under-pin both existing and emerging technologies.

The aim of this study was to evaluate the repeatability of three different real-time PCR platforms including the effect of PCR reaction mix. Previous work carried out has shown that fluorogenic 5' exonuclease ("TaqMan^®^") probe chemistry was more repeatable than Scorpion probe or SYBR^® ^Green I chemistries for genetically modified soya amplification on the LightCycler^® ^(Roche Diagnostics, Lewes, UK) and the ABI PRISM^® ^7700 (Applied Biosystems, Warrington, UK) [13]. Hence, for the amplification of a 'model' plasmid dilution series we have selected "TaqMan^®^" probes to assess inter-platform repeatability of the LightCycler^®^, the ABI PRISM^® ^7700 and the Rotor Gene 3000™ (Corbett Research, Cambridge, UK). These three real-time PCR platforms have different temperature control mechanisms, specifically a 96 well heating block (ABI PRISM^® ^7700), an air fan and/or coil with 32-position carousel (LightCycler^®^), and ambient air with 72-position rotor (Rotor Gene 3000™) [10].

In addition the performance of two commercially available master-mixes were compared. Statistical analysis was performed on the data to assess the levels of inter-platform repeatability and dynamic range of the instruments with different master-mixes.

## Results

### Repeatability Estimates

Precision estimates were determined based on each platform with PCR reaction mix combination at every plasmid copy number across the three days using ISO guidelines [14]. Precision estimates were calculated as repeatability standard deviations of the Ct (threshold cycle number) values from the amplification of each plasmid dilution. In addition, the mean value and standard deviations of these repeatability standard deviations were also presented in Table 1 to provide a measure of the variance of these estimates. Percentage coefficients of variance (%CV) were not used to represent precision estimates as a low mean Ct value in this experiment is indicative of good precision and therefore percentage CV would generate misleading results.

Although the assay was not designed to assess biological sensitivity of the system, and the dilution levels did not approach the limit of detection for the system, the 10^8 ^plasmid copy number data set had the best overall precision associated with it. The most probable reason for this is the availability of the target analyte in the sample.

The ABI PRISM^® ^7700 had the lowest mean repeatability estimate of all the platforms in this study when combined with the FastStart reaction mix, as shown in Table 1. However, the means of the repeatability estimates for the two PCR reaction mixes when combined with this platform were different (0.202 for the FastStart mix and 0.362 for the Excite™ mix). With the exception of the 10^4 ^plasmid copy number, every repeatability estimate value for the reactions performed on the ABI PRISM^® ^7700 using FastStart was lower than the corresponding Excite™ reaction mix. Indeed, consistently across the three platforms, the FastStart mix had the lowest average repeatability estimates in this trial. The value of the mean and standard deviations of the repeatability estimates were comparable when the FastStart PCR reaction mix was employed on the ABI PRISM^® ^7700 and on the LightCycler^® ^(0.202 and 0.219 respectively and 0.047 and 0.082 respectively).

The lowest repeatability estimate calculated in this study was found to be 0.100. This value was generated on the LightCycler^® ^using the FastStart reaction mix and a plasmid copy number of 10^4^. Conversely, the highest repeatability estimate (1.378) was also produced on the LightCycler^® ^but the Excite™ PCR reaction mix was employed with a plasmid copy number of 10^6^.

Unexpectedly, the highest repeatability estimates for each data set are not necessarily associated with the low plasmid copy numbers [15]. The lowest repeatability estimates and the highest repeatability estimates for each platform and PCR reaction mix data set show no discernible trend with respect to plasmid copy number.

The least repeatable system in this trial based upon the data in Table 1 was the combination of the LightCycler^® ^with the Excite™ PCR reaction mix. Comparison with the results of other published studies was not possible, as the statistical methods used were not clearly reported in the literature.

### Analysis of Variance

An ANOVA approach using the threshold cycle number was used to identify sources of variability within the data. Table 2 shows the significance levels of the four main factors of day, plasmid copy number, reaction mix, and platform, as well as the significance levels of the interaction terms. The factors and interaction terms were compared to the Error MS (mean square) in order to assess their significance.

All four individual factors were highly significant. From the calculated F-variance ratio the factor of day had the least significant effect of the four factors, followed by PCR reaction mix (approximately 1.5 times the effect of day), then platform (approximately 7 times the effect of day), and then, as expected, plasmid copy number (approximately 45 times the effect of day). Even though the effect of day had a significant effect upon the results, this effect was very small when compared to the effect of platform and plasmid copy number.

The interaction of the four main factors was also investigated, this demonstrated that the majority of interaction terms were significant. However, in general the magnitude of the effect of these interaction terms was very small compared to the effect of the four main factors.

#### Day Effect

Day had a statistically significant effect upon the Ct value of the raw data (P<0.00001). The average Ct value in the full data set decreased from 19.76 to 18.54 across the 3 days (data not shown).

#### Plasmid Dilution Effect

**Figure 1:**
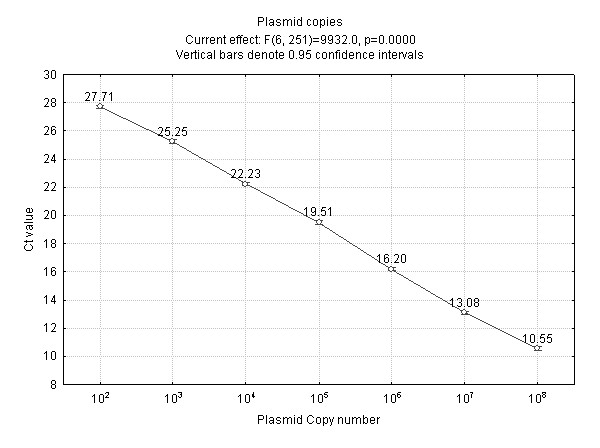
**Average threshold cycle number (Ct) plotted against plasmid copy number**. The data have been pooled from each platform, PCR reaction mix and day to give an average Ct value. Linear regression performed on the ploted data values from this graph determined that the gradient was -2.92 and that the amplification efficiency [21] was greater than two. However the amplification efficiency cannot be greater than two since PCR is an exponential reaction where the amount of product doubles at each cycle.

As Figure 1 illustrates, the number of plasmid copies was inversely proportional to the average Ct value [6,12] within the instrument dynamic working range and above the LOD (limit of detection). Our results concur with Sanchez *et al *[5] who reported that cytomegalovirus quantification was linear between 10^1 ^and 10^6 ^copies, and with Moberg *et al *who used 10^2 ^to 10^7 ^DNA copies in their real-time assay [16]. At extremely low or high plasmid copy numbers the linear relationship with Ct value may be affected. At high copy number it may be difficult to set an accurate baseline because threshold values will be reached after only a few amplification cycles. In addition, reactions with very low numbers of target molecules may be disproportionately affected by pipetting errors and by stoichiometric effects. This highlights the need to work within the linear dynamic range of the instrument and the potential inaccuracy introduced by extrapolation of calibration curves.

Fresh plasmid dilutions were utilised to counteract the previously observed progressive loss of intact plasmid DNA, particularly at low plasmid copy number dilutions. This is probably due to physical adhesion of the DNA to the tube walls, although precautions, such as the use of siliconised tubes, were undertaken.

**Figure 2:**
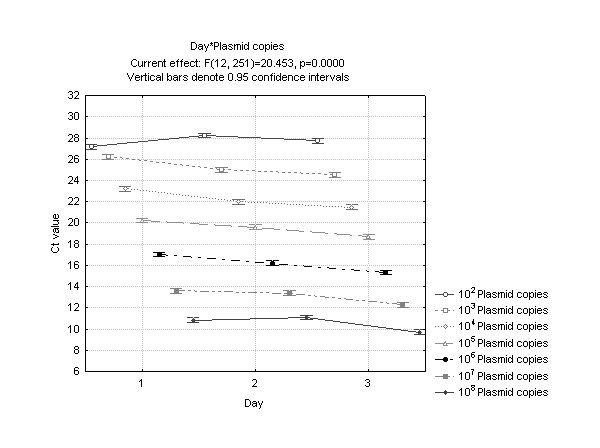
**Average threshold cycle number (Ct) plotted against day for each plasmid dilution illustrating the interaction of day and plasmid copies**. The data have been pooled from each platform, PCR reaction mix and plasmid dilution.

Fresh plasmid dilutions were made each day. Figure 2 illustrates how the average Ct value alters across the three days (which have three different dilution sets) for each plasmid copy number.

#### PCR Reaction Mix Effect

The results from reactions containing FastStart mix had significantly lower average Ct values than their Excite™ 2x mix reaction counterparts (data not presented) – the difference is approximately 1 cycle per plasmid dilution. The smallest interaction term is that of PCR reaction mix with platform (P = 0.019), indicating good consistency of results when using the reaction mixes across different platforms.

#### Platform Effect

**Figure 3:**
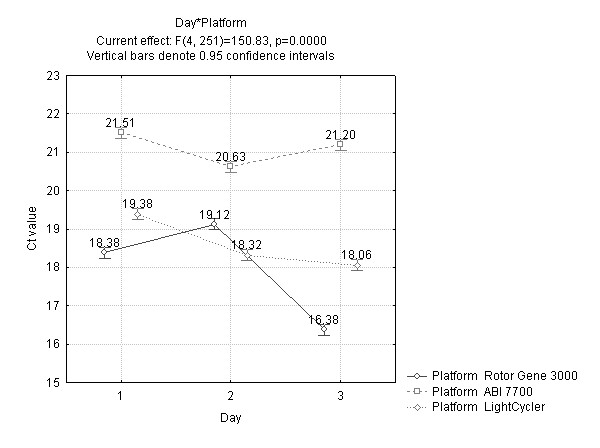
**Average threshold cycle number (Ct) plotted against day for each platform illustrating the interaction of day and platform**. The data have been pooled from each PCR reaction mix and plasmid dilution.

The platforms have different heating mechanisms and therefore it was expected that their performance characteristics such as repeatability might differ significantly. The largest significant interaction term (F = 150.8) was that of platform with day, indicating that each platform may not be behaving consistently between the three days. Closer inspection of the data shows that the majority of the variation associated with the platform by day interaction is attributed to the Rotor Gene day 2, as illustrated in Figure 3, which appears inconsistent compared to the other two platforms. This interaction effect of platform with day was not significantly different from the factor of day or PCR reaction mix. The interaction of PCR reaction mix and platform (shown in Table 2) was found to be statistically significant at the 5% level.

The full raw data set is available [see Additional file 1].

## Discussion

Use of a 'model' system that contained little or no contaminants, coupled with a balanced experimental design enabled accurate assessment of repeatability. Other factors such as extraction methods and complex matrices can potentially increase analytical variability. As demonstrated by the present study, a range of factors can affect performance of each of the instruments. In addition, results are influenced by the threshold value settings applied to the machine by the analyst. To underpin meaningful quantitative PCR measurement, internationally accepted guidelines should be established to standardise the analysis and interpretation of real-time PCR results. PCR reference standards could also be used as indicators of instrument reliability and amplification efficiency [17,18], while internal reaction controls could additionally monitor effects of PCR inhibitors and normalise for any instrument non-uniformity. A recent publication has looked at the plateau phase and the effect of enzyme inhibition during this stage of amplification [19]. With regard to analysis and software compatibility, a study conducted by Kuhne and Oschmann [20] has demonstrated the feasibility of importing standard curves from different LightCycler runs. Historically, standard curves have been the method of choice for determining absolute DNA concentration. However, the quantity of the standards used should be accurately calculated by gravimetry or other primary methods, as absorbance at a wavelength of 260 nm is not sufficient alone. Our present study has shown the potential inaccuracies introduced by importing standard curves, considering the significance of the factor 'day' on the data presented. Even under the controlled conditions used in the present study, significant variability was observed, suggesting that transferring standard curves between runs could be unreliable unless adequate controls are used. Recent research using more sophisticated software approaches has demonstrated the ability to use grand mean calibration curves between runs following compensation for differences in amplification efficiency [21]. Amplification efficiency changes throughout the course of a PCR reaction and is dependent on enzyme kinetics [22,23]. The potential pitfall of assuming that the sample analyte has the exact same kinetics and amplification efficiency of the standards has been highlighted recently [15]. A Galton-Watson branching process has been proposed which involves considering the number of molecules present during PCR cycles and uses Michaelis-Menten enzyme kinetics together with a population growth approach [23]. One other source of uncertainty associated with using amplification curves is the issue of handling outlier data points. In a recent publication Bar *et al *discussed this subject and presented a new statistical method [24]. This may have applications for clinical samples that have been extracted from complex matrices or any analysis with high levels of variability between results. It is not surprising that difficulties in data comparison arise due to the many different mathematical and software approaches applied to real-time quantitative PCR, despite sharing the same mathematical fundamentals [25-28].

The data generated in this study is not intended to indicate any preference for a particular piece of equipment or PCR reaction mix. The instrument specification, reliability, operator training, servicing and comparability all contribute to overall assay precision. In choosing a real-time platform, we would recommend that users consider the statistical outcomes of our results and perform their own critical assessments to ensure that an instrument is fit for their purpose. Generally any such judgement would involve factors such as the volume of samples for processing, the speed at which results need to be obtained and the reproducibility or repeatability of the results.

In addition to choice of appropriate real-time platform, accurate quantitative measurement is dependent upon rational experimental design, optimisation and careful data interpretation.

## Conclusion

We have demonstrated that in this trial the most repeatable system was the ABI PRISM^® ^7700 with the FastStart PCR reaction mix. However the mean repeatability estimate of the LightCycler^® ^combined with the FastStart mix was comparable. In addition, we have shown that the plasmid copy numbers used in this trial did not display a distinct relationship with instrument repeatability.

An ANOVA established that plasmid copy number, platform, PCR reaction mix and day significantly affected the results in a descending order of magnitude.

Inherent differences in the data handling between instruments still presents a challenge to the normalisation of real-time PCR assays between laboratories and users of different instruments. We have confirmed that standardised real-time PCR detection instruments and data analysis approaches are imperative in order to guarantee the integrity and the repeatability of DNA measurement experiments.

## Methods

### Plasmid Composition and Dilution

The target DNA was a pPCR-Script Amp SK(+) plasmid (Stratagene, Amsterdam, The Netherlands) with an insert at the *Srf *I site in the multiple cloning site (supplied by N.I.S.T, Gaithersburg, MD, USA). A portion of the insert sequence follows with the primer binding sites indicated in bold face type and the probe binding site underlined: 5'-***CAGACCGCTCGACGATAGG***TCAGCACTGTCTCGTTGACAGGCGTGGTCAATCAGCCTGAAATCCTCGATCAGAGTGTGC*CG****ATCTCTGGTCCACGTCCT***-3'. From a stock solution of plasmid (3 × 10^13 ^copies) in molecular biology grade water (Sigma, Poole, UK), 1 in 10 serial dilutions were carried out to a copy number of 3 × 10^5 ^per mL using the same batch of water. Fresh dilutions were made every day from the same stock and using the same conditions and were kept at 4°C before and between use. At low copy numbers particularly, a reduction in target copies available for amplification was observed on prolonged storage, possibly due to DNA absorption to the walls of the tube (data not shown). Fresh dilutions were prepared each day in order to minimise this source of variation.

### Real-time PCR Conditions

A 100-bp region of the insert sequence was amplified using HPLC purified oligonucleotide primers (Sigma-Genosys, Cambridge, UK) and a fluorogenic 5' exonuclease probe (Sigma-Genosys, Cambridge, UK) diluted in molecular biology grade water (Sigma, Poole, UK). The primers and probe used were P1A (forward primer): 5'-ACAGACCGCTCGACGATAGG-3', P1B (reverse primer): 5'-AGGACGTGGACCAGAGATCG-3' and TAQ1 (probe): 5'-{FAM}CAGCACTGTCTCGTTGACAGGCGTG{TAMRA}-3' (FAM – 6-carboxyfluoresceine; TAMRA – 5'-carboxytetramethylrhodamine).

Primer Express^® ^software (Applied Biosystems; Warrington, UK) was used to design the primers and probe. The thermal cycling conditions used were dependent on the PCR reaction mix employed and were optimised protocols based on the manufacturer's guidelines. Amplification conditions for those reactions that contained 'FastStart Master mix' (Roche Diagnostics, Lewes, UK) were a 10 minute hold at 95°C followed by 55 cycles of 95°C for 15 seconds and 60°C for 30 seconds. For reactions with the Excite™ 2x kit (Biogene, Kimbolton, UK) the amplification conditions were a 2 minute hold at 50°C followed by a 10 minute hold at 95°C and then 55 cycles of 95°C for 15 seconds and 60°C for one minute.

### Instrumentation & PCR Reaction Mixes

Three platforms compatible with "TaqMan^®^" probes were assessed – LightCycler^® ^(Roche Diagnostics, Lewes, UK), ABI PRISM^® ^7700 (Applied Biosystems, Warrington, UK) and Rotor Gene 3000™ (Corbett Research, Cambridge, UK). All of these instruments were found to be compatible with FastStart (Roche Diagnostics, Lewes, UK) and Excite™ 2x (Biogene Kimbolton, UK) mixes following optimisation. The amplification performances of both PCR reaction mixes were investigated on each of the three machines using the plasmid dilution series as target. The probe was added at a final concentration of 200 nM per reaction for all experiments. The Primers P1A and P1B were used at a final concentration of 1500 nM each in the Excite™ mix reactions whereas the FastStart mix reactions contained the primers at a final concentration of 500 nM each. A supplement of magnesium chloride (final concentration of 3 mM) was added to those reactions containing Excite™ mix that were run on the LightCycler^®^. In addition, magnesium chloride at a final concentration of 3 mM was required for all those reactions containing the FastStart mix except for the equivalent amplification reactions run on the LightCycler^®^, where a final concentration of 2 mM of magnesium chloride was optimal.

A solution of BSA (BSA 100x; New England Biolabs, Hitchin, UK) at a final concentration of 0.1 mg/mL was added to the Excite mix for reactions run on the LightCycler^®^. To every reaction except the negative controls, 1 μL of plasmid DNA was added. Additional molecular biology grade water was added to a final volume of 20 μL and 25 μL for the FastStart and Excite™ mixes respectively.

Reactions were set up in 20 μL glass capillaries, 0.1 mL plastic tubes and 96 well optical plates for use on the LightCycler^®^, Rotor Gene 3000™ and ABI PRISM^® ^7700 respectively.

### Experimental Design

Six experiments (two per platform) were run, and repeated for three consecutive days. Three replicates of each plasmid DNA dilution plus three negative controls (24 reactions in total) were amplified per experiment. One specific analyst prepared the complete master-mixes containing primers, probes, water and other supplements as already outlined, in a pre-PCR 'clean' room using the same pipettes each day. A second specific analyst added the plasmid DNA in a dedicated template addition area and used the same instruments for all template additions. Those amplification reactions containing the Excite™ 2x mix were run at the same time each morning on the Rotor Gene 3000™, ABI PRISM^® ^7700 and LightCycler^® ^and the reactions containing FastStart mix were run at the same time each afternoon on the Rotor Gene 3000™, ABI PRISM^® ^7700 and LightCycler^®^. For each run the same instrument, rotor and carousel (where applicable) were employed to minimise intra-run variability.

### Baseline Settings & Data

The Ct (threshold cycle number) was used as the measurand in order to assess the level of product in proportion to the level of starting target. As recommended by the manufacturers' guidelines, the Ct values were calculated from the mid-point of the exponential phase of amplification. However, each real-time platform required some manual intervention to set analysis parameters. In addition specific analysis software was associated with each instrument and inherent differences in data manipulation may introduce further inter-platform variability. As amplification efficiency has been shown to change over the course of the reaction [29], we have based our analysis on the mid-point of the exponential phase to measure threshold cycles irrespective of platform, run or PCR reaction mix.

### Statistical Analysis

The results were interpreted and analysed according to the instrument manufacturer protocols. For each platform, graphs of cycle number versus arbitrary fluorescence units were generated and from this raw data, Ct values were automatically calculated by the software provided with each instrument [see Additional file 1]. The statistical package Statistica 6.0 (Statsoft; Tulsa, OK, USA) was used to analyse the data for all the instrument runs by ANOVA (Analysis of Variance) and then repeatability estimates were carried out.

## Abbreviations

FAM: 6-carboxyfluoresceine

TAMRA: 5-carboxytetramethylrhodamine

BSA: bovine serum albumin

Ct: threshold cycle number

%CV: Percentage coefficient of variance

MS: Mean square

## Authors' contributions

CED carried out the experimental work and drafted the manuscript; FQ carried out the experimental work; MJB participated in the design of the study and performed the statistical analysis; MJH conceived of the study; JRB produced the plasmid material; AJW conceived and designed the study. All authors read and approved the final manuscript.

## Supplementary Material

Additional File 1 **Raw Data Table**. Table 3 consists of the raw data results comparing the effect of day, plasmid copy number, PCR reaction mix and platform on the mean Ct value. Click here for file

## Figures and Tables

**Table 1:** Comparison of repeatability estimates of the PCR measurements using different reaction mixes and platforms over three days (using standard deviations of the Ct value).

Repeatability Estimates (standard deviations of the Ct value) based on Individual Plasmid Copy Number						
Plasmid Copy Number	Platform and PCR Reaction Mix					
	
	ABI 7700		LightCycler		RotorGene	
	
	Excite	FastStart	Excite	FastStart	Excite	FastStart

10^2^	0.543	0.182	0.568	0.233	0.888	0.186
10^3^	0.452	0.204	1.177	0.190	0.184	0.445
10^4^	0.223	0.305	1.123	0.100	0.256	0.592
10^5^	0.243	0.168	0.947	0.224	0.209	0.221
10^6^	0.362	0.183	1.378	0.281	0.772	0.333
10^7^	0.473	0.189	0.919	0.156	0.319	0.247
10^8^	0.236	0.184	0.454	0.352	0.322	0.166
Degrees of Freedom	6–8	6	6–8	5–6	6–8	6
Mean	0.362	0.202	0.938	0.219	0.421	0.313
Standard Deviation	0.130	0.047	0.331	0.082	0.286	0.156

(Note that one replicate is missing for the LC platform coupled with the Fast Start reaction mix – the capillary broke).

**Table 2:** Variation attributable to day, plasmid copy number, PCR reaction mix and platform following an ANOVA test using threshold cycle number (Ct) as the variable factor.

	**Univariate Tests of Significance for Ct value. Sigma-restricted parameterisation. Effective hypothesis decomposition.**				
**Effect**	**SS**	**DF**	**MS**	**F**	**P**

Day	96.0	2	48.0	221.4	<0.00001
Plasmid Copies	12919.2	6	2153.2	9932.0	<0.00001
PCR Reaction Mix	75.0	1	75.0	346.2	<0.00001
Platform	700.5	2	350.2	1615.5	<0.00001
Day with Plasmid Copies	53.2	12	4.4	20.5	<0.00001
Day with PCR Reaction Mix	1.9	2	0.9	4.3	0.01442
Plasmid Copies with PCR Reaction Mix	8.5	6	1.4	6.5	<0.00001
Day with Platform	130.8	4	32.7	150.8	<0.00001
Plasmid Copies with Platform	51.0	12	4.2	19.6	<0.00001
PCR Reaction Mix with Platform	1.7	2	0.9	4.0	0.01901
Day with Plasmid Copies with PCR Reaction Mix	14.6	12	1.2	5.6	<0.00001
Day with Plasmid copies with Platform	18.3	24	0.8	3.5	<0.00001
Day with PCR Reaction Mix with Platform	49.6	4	12.4	57.1	<0.00001
Plasmid copies with PCR Reaction Mix with Platform	4.5	12	0.4	1.7	0.06337
Day with Plasmid Copies with PCR Reaction Mix with Platform	11.8	24	0.5	2.3	0.00091
Error	54.4	251	0.2		

(Where SS = sum of squares; DF = degrees of freedom; MS = Mean Square; F = ratio of variances; p = probability of obtaining a specific result, given by the null hypothesis).
