# Supplementary material for: An inter-platform repeatability study investigating real-time amplification of plasmid DNA
Source: BMC Biotechnol. 2005 May 25;5:15. doi: 10.1186/1472-6750-5-15 (PMC1168890; doi:10.1186/1472-6750-5-15)
Supplement: Additional File 1 — Raw Data Table. Table 3 consists of the raw data results comparing the effect of day, plasmid copy number, PCR reaction mix and platform on the mean Ct value. [file 1472-6750-5-15-S1.doc]

Table 3: Raw data results comparing the effect of day, plasmid copy number, PCR reaction mix and platform on the mean Ct value (Supplementary data). Where ABI 7700 represents the ABI PRISM 7700.

| **Day** | **Plasmid Copy number** | **PCR Reaction Mix** | **Platform** | **Mean Ct value** |
| --- | --- | --- | --- | --- |
| 1 | 10^2^ | Excite | Rotor Gene 3000 | 25.343 |
| 1 | 10^3^ | Excite | Rotor Gene 3000 | 24.247 |
| 1 | 10^4^ | Excite | Rotor Gene 3000 | 21.857 |
| 1 | 10^5^ | Excite | Rotor Gene 3000 | 18.630 |
| 1 | 10^6^ | Excite | Rotor Gene 3000 | 14.917 |
| 1 | 10^7^ | Excite | Rotor Gene 3000 | 11.917 |
| 1 | 10^8^ | Excite | Rotor Gene 3000 | 9.053 |
| 1 | 10^2^ | Excite | ABI 7700 | 30.180 |
| 1 | 10^3^ | Excite | ABI 7700 | 28.723 |
| 1 | 10^4^ | Excite | ABI 7700 | 25.657 |
| 1 | 10^5^ | Excite | ABI 7700 | 22.090 |
| 1 | 10^6^ | Excite | ABI 7700 | 18.997 |
| 1 | 10^7^ | Excite | ABI 7700 | 16.000 |
| 1 | 10^8^ | Excite | ABI 7700 | 12.977 |
| 1 | 10^2^ | Excite | LightCycler | 27.707 |
| 1 | 10^3^ | Excite | LightCycler | 26.410 |
| 1 | 10^4^ | Excite | LightCycler | 22.873 |
| 1 | 10^5^ | Excite | LightCycler | 20.133 |
| 1 | 10^6^ | Excite | LightCycler | 19.993 |
| 1 | 10^7^ | Excite | LightCycler | 13.823 |
| 1 | 10^8^ | Excite | LightCycler | 10.640 |
| 1 | 10^2^ | FastStart | Rotor Gene 3000 | 24.643 |
| 1 | 10^3^ | FastStart | Rotor Gene 3000 | 25.030 |
| 1 | 10^4^ | FastStart | Rotor Gene 3000 | 22.410 |
| 1 | 10^5^ | FastStart | Rotor Gene 3000 | 20.117 |
| 1 | 10^6^ | FastStart | Rotor Gene 3000 | 15.457 |
| 1 | 10^7^ | FastStart | Rotor Gene 3000 | 12.883 |
| 1 | 10^8^ | FastStart | Rotor Gene 3000 | 10.773 |
| 1 | 10^2^ | FastStart | ABI 7700 | 29.043 |
| 1 | 10^3^ | FastStart | ABI 7700 | 27.413 |
| 1 | 10^4^ | FastStart | ABI 7700 | 24.260 |
| 1 | 10^5^ | FastStart | ABI 7700 | 21.153 |
| 1 | 10^6^ | FastStart | ABI 7700 | 17.270 |
| 1 | 10^7^ | FastStart | ABI 7700 | 14.847 |
| 1 | 10^8^ | FastStart | ABI 7700 | 12.497 |
| 1 | 10^2^ | FastStart | LightCycler | 26.050 |
| 1 | 10^3^ | FastStart | LightCycler | 25.523 |
| 1 | 10^4^ | FastStart | LightCycler | 22.193 |
| 1 | 10^5^ | FastStart | LightCycler | 19.303 |
| 1 | 10^6^ | FastStart | LightCycler | 15.500 |
| 1 | 10^7^ | FastStart | LightCycler | 12.073 |
| 1 | 10^8^ | FastStart | LightCycler | 9.150 |
| 2 | 10^2^ | Excite | Rotor Gene 3000 | 26.417 |
| 2 | 10^3^ | Excite | Rotor Gene 3000 | 24.830 |
| 2 | 10^4^ | Excite | Rotor Gene 3000 | 23.067 |
| 2 | 10^5^ | Excite | Rotor Gene 3000 | 20.613 |
| 2 | 10^6^ | Excite | Rotor Gene 3000 | 17.173 |
| 2 | 10^7^ | Excite | Rotor Gene 3000 | 15.197 |
| 2 | 10^8^ | Excite | Rotor Gene 3000 | 12.310 |
| 2 | 10^2^ | Excite | ABI 7700 | 29.927 |
| 2 | 10^3^ | Excite | ABI 7700 | 26.933 |
| 2 | 10^4^ | Excite | ABI 7700 | 24.127 |
| 2 | 10^5^ | Excite | ABI 7700 | 21.460 |
| 2 | 10^6^ | Excite | ABI 7700 | 18.080 |
| 2 | 10^7^ | Excite | ABI 7700 | 14.723 |
| 2 | 10^8^ | Excite | ABI 7700 | 12.653 |
| 2 | 10^2^ | Excite | LightCycler | 28.023 |
| 2 | 10^3^ | Excite | LightCycler | 24.477 |
| 2 | 10^4^ | Excite | LightCycler | 20.837 |
| 2 | 10^5^ | Excite | LightCycler | 18.577 |
| 2 | 10^6^ | Excite | LightCycler | 15.367 |
| 2 | 10^7^ | Excite | LightCycler | 12.473 |
| 2 | 10^8^ | Excite | LightCycler | 9.705 |
| 2 | 10^2^ | FastStart | Rotor Gene 3000 | 26.557 |
| 2 | 10^3^ | FastStart | Rotor Gene 3000 | 23.483 |
| 2 | 10^4^ | FastStart | Rotor Gene 3000 | 20.527 |
| 2 | 10^5^ | FastStart | Rotor Gene 3000 | 19.203 |
| 2 | 10^6^ | FastStart | Rotor Gene 3000 | 15.107 |
| 2 | 10^7^ | FastStart | Rotor Gene 3000 | 12.380 |
| 2 | 10^8^ | FastStart | Rotor Gene 3000 | 10.827 |
| 2 | 10^2^ | FastStart | ABI 7700 | 30.163 |
| 2 | 10^3^ | FastStart | ABI 7700 | 26.157 |
| 2 | 10^4^ | FastStart | ABI 7700 | 22.487 |
| 2 | 10^5^ | FastStart | ABI 7700 | 19.953 |
| 2 | 10^6^ | FastStart | ABI 7700 | 16.533 |
| 2 | 10^7^ | FastStart | ABI 7700 | 13.743 |
| 2 | 10^8^ | FastStart | ABI 7700 | 11.827 |
| 2 | 10^2^ | FastStart | LightCycler | 28.300 |
| 2 | 10^3^ | FastStart | LightCycler | 24.060 |
| 2 | 10^4^ | FastStart | LightCycler | 20.873 |
| 2 | 10^5^ | FastStart | LightCycler | 17.760 |
| 2 | 10^6^ | FastStart | LightCycler | 15.090 |
| 2 | 10^7^ | FastStart | LightCycler | 11.760 |
| 2 | 10^8^ | FastStart | LightCycler | 9.126 |
| 3 | 10^2^ | Excite | Rotor Gene 3000 | 25.490 |
| 3 | 10^3^ | Excite | Rotor Gene 3000 | 23.050 |
| 3 | 10^4^ | Excite | Rotor Gene 3000 | 20.277 |
| 3 | 10^5^ | Excite | Rotor Gene 3000 | 17.300 |
| 3 | 10^6^ | Excite | Rotor Gene 3000 | 14.373 |
| 3 | 10^7^ | Excite | Rotor Gene 3000 | 10.957 |
| 3 | 10^8^ | Excite | Rotor Gene 3000 | 8.277 |
| 3 | 10^2^ | Excite | ABI 7700 | 30.653 |
| 3 | 10^3^ | Excite | ABI 7700 | 27.643 |
| 3 | 10^4^ | Excite | ABI 7700 | 24.540 |
| 3 | 10^5^ | Excite | ABI 7700 | 21.317 |
| 3 | 10^6^ | Excite | ABI 7700 | 17.933 |
| 3 | 10^7^ | Excite | ABI 7700 | 14.993 |
| 3 | 10^8^ | Excite | ABI 7700 | 12.297 |
| 3 | 10^2^ | Excite | LightCycler | 27.923 |
| 3 | 10^3^ | Excite | LightCycler | 24.960 |
| 3 | 10^4^ | Excite | LightCycler | 21.710 |
| 3 | 10^5^ | Excite | LightCycler | 18.730^2^ |
| 3 | 10^6^ | Excite | LightCycler | 15.367 |
| 3 | 10^7^ | Excite | LightCycler | 12.420 |
| 3 | 10^8^ | Excite | LightCycler | 9.533 |
| 3 | 10^2^ | FastStart | Rotor Gene 3000 | 24.383 |
| 3 | 10^3^ | FastStart | Rotor Gene 3000 | 21.077 |
| 3 | 10^4^ | FastStart | Rotor Gene 3000 | 18.493 |
| 3 | 10^5^ | FastStart | Rotor Gene 3000 | 16.007 |
| 3 | 10^6^ | FastStart | Rotor Gene 3000 | 12.580 |
| 3 | 10^7^ | FastStart | Rotor Gene 3000 | 9.703 |
| 3 | 10^8^ | FastStart | Rotor Gene 3000 | 7.357 |
| 3 | 10^2^ | FastStart | ABI 7700 | 30.787 |
| 3 | 10^3^ | FastStart | ABI 7700 | 26.910 |
| 3 | 10^4^ | FastStart | ABI 7700 | 23.827 |
| 3 | 10^5^ | FastStart | ABI 7700 | 21.170 |
| 3 | 10^6^ | FastStart | ABI 7700 | 17.527 |
| 3 | 10^7^ | FastStart | ABI 7700 | 14.620 |
| 3 | 10^8^ | FastStart | ABI 7700 | 12.530 |
| 3 | 10^2^ | FastStart | LightCycler | 27.137 |
| 3 | 10^3^ | FastStart | LightCycler | 23.527 |
| 3 | 10^4^ | FastStart | LightCycler | 20.147 |
| 3 | 10^5^ | FastStart | LightCycler | 17.700 |
| 3 | 10^6^ | FastStart | LightCycler | 14.297 |
| 3 | 10^7^ | FastStart | LightCycler | 11.003 |
| 3 | 10^8^ | FastStart | LightCycler | 8.338 |
